# A Compendium of the Lectures on the Theory and Practice of Medicine, Delivered by Professor Chapman in the University of Pennsylvania

**Published:** 1846-06

**Authors:** 


ART. VII.—*A Compendium of the Lectures on the Theory and Practice of Medicine, delivered by Professor Chapman, in the University of Pennsylvania.* Prepared with permission from Dr. Chapman's manuscripts, and published with his approbation. By N. D. BENEDICT, M.D., Fellow of the College of Physicians of Philadelphia; Physician to the Lying-in Department of the Philadelphia Hospital. Philadelphia:—Lea & Blanchard. 1846. 8vo. pp. 258.

This Compendium embraces none of the matter previously given to the public in the two volumes, by Professor Chapman, on Diseases of the Thoracic and Abdominal Viscera, and on Eruptive Fevers, &c., and is therefore a summary of only a part of the course of lectures delivered by that eminent teacher. The title of the work clearly expresses its character. It contains, in a small compass, the substance of what Dr. Chapman communicates in his lectures; and however little to the advantage of the author it may be to appear before the world in this fashion, it cannot be doubted that the practitioner of medicine will find a great convenience in having the matured experience of an able man thus served up in a nut-shell. We have not room to make quotations from the work; if we could do so, the reader would see that in this compendium he would possess a work, to which he might refer for practical hints on a vast variety of subjects—a '*receipt book*' on medicine, of the highest order of excellence, because emanating from one of the first minds in our profession. Such a compendium ought not to take the place of the elaborate lectures of which it purports to be the cream; but there is an office which it is well fitted to perform; and, as a remembrancer to the author's many pupils, and a book of easy reference for the practising physician, it is likely to be well received.

